# Dopamine in the Regulation of Glucose Homeostasis, Pathogenesis of Type 2 Diabetes, and Chronic Conditions of Impaired Dopamine Activity/Metabolism: Implication for Pathophysiological and Therapeutic Purposes

**DOI:** 10.3390/biomedicines11112993

**Published:** 2023-11-07

**Authors:** Giuseppe Lisco, Anna De Tullio, Michele Iovino, Olga Disoteo, Edoardo Guastamacchia, Vito Angelo Giagulli, Vincenzo Triggiani

**Affiliations:** 1 Interdisciplinary Department of Medicine, School of Medicine, University of Bari, 70124 Bari, Italy; giuseppe.lisco@uniba.it (G.L.); annadetullio16@gmail.com (A.D.T.); micheleiovino06@libero.it (M.I.); edoardo.guastamacchia@uniba.it (E.G.); vitogiagulli58@gmail.com (V.A.G.); 2 Diabetology Unit, ASST Grande Ospedale Metropolitano Niguarda, 20162 Milan, Italy; olgaeugenia.disoteo.amediabete@gmail.com

**Keywords:** dopamine, levodopa, type 2 diabetes mellitus, insulin, glucagon-like receptor 1, incretin system, Parkinson’s disease, ADHD

## Abstract

Dopamine regulates several functions, such as voluntary movements, spatial memory, motivation, sleep, arousal, feeding, immune function, maternal behaviors, and lactation. Less clear is the role of dopamine in the pathophysiology of type 2 diabetes mellitus (T2D) and chronic complications and conditions frequently associated with it. This review summarizes recent evidence on the role of dopamine in regulating insular metabolism and activity, the pathophysiology of traditional chronic complications associated with T2D, the pathophysiological interconnection between T2D and chronic neurological and psychiatric disorders characterized by impaired dopamine activity/metabolism, and therapeutic implications. Reinforcing dopamine signaling is therapeutic in T2D, especially in patients with dopamine-related disorders, such as Parkinson’s and Huntington’s diseases, addictions, and attention-deficit/hyperactivity disorder. On the other hand, although specific trials are probably needed, certain medications approved for T2D (e.g., metformin, pioglitazone, incretin-based therapy, and gliflozins) may have a therapeutic role in such dopamine-related disorders due to anti-inflammatory and anti-oxidative effects, improvement in insulin signaling, neuroinflammation, mitochondrial dysfunction, autophagy, and apoptosis, restoration of striatal dopamine synthesis, and modulation of dopamine signaling associated with reward and hedonic eating. Last, targeting dopamine metabolism could have the potential for diagnostic and therapeutic purposes in chronic diabetes-related complications, such as diabetic retinopathy.

## 1. Background

Dopamine has several systemic effects and may have a role in the pathophysiology of chronic diseases. This review aims to summarize the leading evidence regarding the pathophysiological and potentially therapeutic role of dopamine in type 2 diabetes (T2D) and to explore the cross-linking between T2D pathophysiology and the most frequent chronic neurologic and psychiatric disorders associated with impaired dopamine activity/metabolism and T2D in human pathology.

Dopamine (C_8_H_11_NO_2_) is a catecholamine derived by the amino acid tyrosine after a two-step reaction involving (a) the enzyme tyrosine hydroxylase (step 1), which transforms the amino acid tyrosine in L-dihydroxyphenylalanine (L-DOPA), and (b) the enzyme DOPA decarboxylase (step 2), which converts L-DOPA in dopamine [1]. Dopamine is also the precursor of noradrenaline (dopamine β-hydroxylase) and adrenaline (phenyl-ethanolamine N-methyltransferase) [2].

Dopamine is synthesized mainly in the brain and other tissues with neuroectodermal origin, such as the medulla of the adrenal glands and paraganglia, where it serves as the precursor of adrenaline and noradrenaline synthesis. Dopamine is well known as a neurotransmitter, but extracerebral sites of production and dopamine receptors (DRs) are also widely expressed in peripheral tissues.

**Figure 1:**
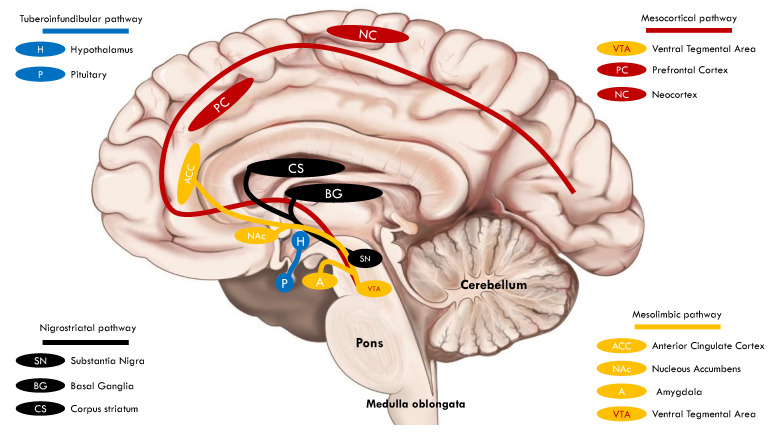
Simplified depiction of the four dopaminergic pathways in the central nervous system.

Four dopaminergic pathways exist in the central nervous system: the mesolimbic, mesocortical, nigrostriatal, and tuberoinfundibular (Figure 1). In the mesolimbic pathway, dopaminergic neurons of the ventral tegmental area (VTA) project to the nucleus accumbens (NAc), anterior cingulate cortex, and amygdala (AMY). The pathway involves pleasure and reward, and its dysfunction is associated with neuropsychiatric disorders such as schizophrenia, depression, and chronic pain [3]. Dopaminergic projections within the mesocortical pathway also originate in the VTA. The firing of VTA dopaminergic neurons travels to some areas in the prefrontal cortex (PC), which regulates critical cognitive functions such as cognition, working memory, and decision making [4]. The nigrostriatal pathway is the foremost dopaminergic system in the brain and is involved in controlling voluntary movement. Dopamine projections start in the substantia nigra and fire to the caudate and putamen in the basal ganglia. This dopaminergic pathway is affected in some neurological disorders involving the extrapyramidal system, such as Parkinson’s disease (PD), and in patients chronically treated with first-generation antipsychotics (D_2_ receptor antagonists), resulting in irregular muscle contractions manifesting as tremors, spasms, motor restlessness, and tardive dyskinesia. In the tuberoinfundibular pathway, dopamine neurons begin in the hypothalamic arcuate and periventricular nuclei and project to the median eminence of the hypothalamus. After that, dopamine is released into the portal circulation, connecting the median eminence to the pituitary gland, where dopamine inhibits the prolactin release from lactotrophic cells. A mild or moderate increase in serum prolactin is a secondary effect of D_2_ antagonists [5].

## 2. The Effects of Dopamine on Pancreatic Islets and Insulin and Glucagon Secretion

The potential role of endogenous catecholamines in the pathogenesis of T2D was suggested by landmark studies in the 1970s [6,7,8]. The intravenous administration of L-DOPA increased the pancreatic dopamine concentration, especially within the β-cells, in normal rats [9] and inhibited insulin secretion in several species of golden hamsters [10]. A mechanistic study found that intravenous administration of L-DOPA was accompanied by a subsequent increase in the dopamine-containing grains in β-cells. Accumulation of dopamine-containing grains was found to reduce the release of insulin-containing grains by secretagogues, ultimately indicating that dopamine partially suppressed insulin release from β-cells [11]. Another study confirmed that dopamine suppresses insulin release from β-cells. The dopamine effect was completely inverted after the administration of propranolol (a β-blocker) but was not affected by dopamine antagonists, indicating that the suppression of insulin release by dopamine was mediated by α-adrenergic rather than dopaminergic signaling [12]. In an obese murine model (ob/ob), dopaminergic therapy reduced hyperglycemia and hyperlipidemia and improved islet function by restoring glucose sensitivity in β-cells (assessed by a 1.6-fold increase in the Glucokinase immunoreactivity), stabilizing hyperplasia, enhancing insulin storage, and thus reducing circulating insulin levels [13]. A recent investigation demonstrated that pancreatic islets are a site of dopamine synthesis and that L-DOPA and dopamine reduce glucose-dependent insulin secretion by dropping the frequency of intracellular oscillations of calcium currents. This effect was mediated directly by DR_3_ stimulation, as demonstrated by experiments using specific dopamine antagonists [14]. In another study, a single administration of the dopamine agonist bromocriptine reduced fasting glucose and insulin levels in patients with T2D. These effects were only mild in healthy controls. They were accompanied by a reduction in prolactin levels in all and growth hormone concentrations only in T2D patients, suggesting that the bromocriptine effect on glucose control could largely depend on an insulin-sensitizing secondary impact, mainly due to a reduction in growth hormone levels [15]. Apart from the effect on insulin secretion, the proliferation rate of β-cells decreases, and the apoptosis increases following dopamine treatment [16]. An inverse correlation between circulating levels of dopamine and c-peptide (a biomarker of insulin secretion from β-cells) was demonstrated in 201 healthy voluntaries [17], in which the insulin suppressive effect of dopamine was mediated by both DR_2_ and DR_3_ signaling [18,19]. In a recent study on rodents, dopamine dampened glucose-stimulated insulin secretion after a meal challenge test by counteracting the incretin effect, indicating that dopamine could affect insulin secretion in the post-prandial phase [20]. Glucose intake increases circulating dopamine levels by stimulating the intestinal secretion of dopamine, and this mechanism could work as a brake effect on the incretin actions [21]. As an additional mechanism, dopamine suppresses prolactin secretion. Prolactin stimulates insulin secretion and β-cell proliferation. It plays a role in normal pancreatic development and ameliorates peripheral insulin sensitivity, especially at the level of the adipose tissue [22].

Given the anti-secretive and antiproliferative effects, dopamine may have a role in the pathophysiology of T2D. Monoaminoxidase A and B play a crucial role in the catabolism of catecholamines, including dopamine. Both isoforms are also expressed in β-cells, and a lower level of monoaminoxidase activity is associated with dampened insulin secretion. Therefore, this evidence suggests that blunted dopamine catabolism and, consequently, high intra-islet dopamine concentration may contribute to reducing insulin secretion and raising the number of apoptotic β-cells, both events primarily involved in the pathophysiology of T2D. Interestingly, the transcription of monoaminoxidase A and B genes is under the MAF transcription factor A control [23]. MAF transcription factor A is an essential regulator of β-cell transcriptional activity since it regulates the transcription of genes involved in specific β-cell activities, including insulin biosynthesis and secretion [24]. The activity level and expression of the MAF factor A depend on glucose levels and may be reduced significantly by glucotoxicity due to hyperglycemia and chronic low-grade inflammation observed in prediabetes and diabetes [25]. Experimental models of insulinopenic, such as streptozotocin-induced, diabetes indicated that insulin deficiency increases the activity of circulating dopamine β-hydroxylase (which converts dopamine into noradrenaline), and the administration of insulin significantly reduces the enzymatic activity [26,27]. The phenomenon was associated with an increased dopamine receptor binding (up-regulation) in the striatum [28], which was the probable consequence of reduced dopamine metabolism in the same cerebral area [29,30].

These data suggest that dopamine and insulin may be involved in a potential feedback mechanism in which one negatively regulates the metabolism of the other [31].

Pivotal studies suggested that intravenous dopamine infusion stimulated glucagon release [32] in a dose-dependent manner [33]. Keck et al. found that low-dose dopamine (e.g., 2 mcg/kg/min infused for 6 consecutive hours) did not affect both insulin and glucagon secretion [34], but high-dose dopamine was found to provide relevant hyperglycemia by suppressing insulin and stimulating glucagon secretion in rats and men [32,33]. The effect could be considered an additive mechanism by which dopamine and dopamine agonists could sustain hyperglycemia in healthy and T2D patients. A summary of the mechanisms by which dopamine affects β-cell activity, insulin, and glucagon secretion is shown in Table 1.

## 3. Dopamine in the Pathogenesis and Treatment of Traditional Chronic Diabetes-Related Complications

T2D and chronic comorbidities, such as arterial hypertension, overweight/obesity, and dyslipidemia, foster the development of chronic complications over time [35]. Hyperglycemia is the determinant of chronic diabetes-related complications, especially at the microvascular site, and more stringent glucose control is associated with a lower likelihood of the onset and progression of these complications [36]. Moreover, early intensive intervention to achieve optimal control of all risk factors concomitant with T2D is associated with a reduced risk of macrovascular complications [37], and the higher the stability of glucose control over time, the better the attenuation of burdens [38,39]. So far, guidelines recommend comprehensive management of T2D patients to reduce the risk of diabetes-related complications over time by targeting glucose, arterial pressure, body weight, and lipid control, as well as preventing thrombotic events and attenuating thrombotic risks [40]. Evidence is already consolidated to suggest the use of specific classes of medications, such as glucagon-like peptide 1 receptor agonists (GLP-1RAs) and sodium-glucose (co)transporter 2 inhibitors (SGLT2is), to improve hard clinical outcomes, reduce the risk of adverse cardiovascular and renal endpoints, hospital admission due to heart failure and heart failure progression, and diabetes-related mortality [41,42].

The role of dopamine in the pathophysiology of diabetes-related chronic complications is an emerging issue [43]. Comprehending the mechanisms involved in the physiological activities of dopamine and the pathophysiological disruption of dopamine metabolism and dopaminergic pathways in target tissues would have relevant therapeutic implications and advance current treatments (Table 2). Dopaminergic neurons are described in the retina, where dopamine is a neurotransmitter. Here, dopamine diffuses through retinal layers to reach target cells and modulate their activity. Hence, the mechanism of dopamine communication in the retinal tissue is volume-dependent. In other words, dopamine deficiency or impaired metabolism/activity could be associated with retinal disease [44]. Experimental studies suggest that dopamine regulates photoreceptor activity, critical to visual adaptation to daylight [45]. Intraretinal dopamine levels are low in the early phase of retinal damage in diabetes [46], while high intraretinal levels of dopamine are protective against retinal damage and visual field loss [47]. The precise mechanism by which preserving dopamine levels in retinal tissue would prevent retinal damage and visual impairment is unclear. Experimental models found that intravitreal administration of L-DOPA was associated with lower severity of hyperglycemic memory-induced retinal microvascular alterations, including pericyte degeneration, acellular capillary and pericyte ghost generation, and endothelial apoptosis [48]. One mechanistic study in rodents has recently shown that intravitreal administration of L-DOPA reduced intraretinal levels of the vascular endothelial growth factor and insulin-like growth factor 1 receptors via the AKT/ERK pathway after 12 weeks [49]. Nevertheless, the first data available on a few cases did not confirm relevant differences in intraretinal dopamine (metabolites) in patients with diabetes without clinical signs of diabetic retinopathy and those without diabetes [50]. Additional studies are needed to verify whether intraretinal dopamine metabolism in humans differs from what has been seen in experimental models. On the other hand, the results of a pilot trial confirmed that reinforcing the intraretinal dopamine pathway may improve retinal dysfunction in the early stages of diabetic retinopathy [51]. So far, concrete pathophysiological hypotheses suggest a link between neurodegenerative disease and diabetic retinopathy in T2D [52], and evidence supports the role of diagnostic intervention in the early stages of both diseases [53]. From a therapeutic viewpoint, specific trials are currently ongoing to investigate the role of dopamine replacement in early-stage diabetic retinopathy and diabetic macular edema (NCT05132660; NCT02706977; NCT03161652). GLP-1 agonists may accelerate the progression of diabetic retinopathy and can be associated with adverse retinal outcomes while improving glucose control. Although evidence is discordant, data from the literature reported that this effect could be restricted to only some specific analogs and could be related to some background characteristics, such as poor glycemic control, more rapid achievement of glucose targets, higher body weight, and the presence of very high cardiovascular risk [54,55]. The above results align with preclinical evidence suggesting that GLP-1 analogs promote endothelial cell growth and angiogenesis. It could be interesting to assess the role of GLP-1 analogs on the intraretinal dopaminergic pathway. One trial could clarify this issue (NCT02671864).

Dopamine and DRs in the nephron tubules are essential in regulating key renal functions, such as electrolytes and water resorption, acid–base balance, and blood pressure regulation. DR_1_ and DR_2_ are the most widely expressed receptors mediating dopamine activity in the whole body [56]. In chronic diseases, such as arterial hypertension and diabetes, the expression of dopamine receptors could be significantly impaired in the kidney and the dopamine metabolism altered [57]. Because of these detrimental mechanisms, water exertion and natriuresis can be substantially reduced, thus contributing to water and sodium retention, increased blood pressure, glomerular hyperfiltration, and micro-/macroalbuminuria [58]. Experimental data in rats suggested that high intrarenal levels of dopamine prevent the mentioned effects and protect against glomerular injury and progression of diabetic nephropathy [59]. One pilot study found that administering bromocriptine (a dopamine agonist) compared to placebo reduced blood pressure and the left ventricular mass index without deteriorating the glomerular filtration rate in T2D over 6 months of treatment [60].

Dopamine plays many actions in the human heart, including positive inotropic and chronotropic effects, regulation of coronary flow, and cardiomyocyte metabolism [61]. These effects are mediated directly by dopamine and its interaction with DRs or indirectly by dopamine and noradrenaline action on α-adrenergic receptors [62]. Early evidence suggested the existence of impaired intracardiac dopamine metabolism in patients with diabetes [63]. More recent evidence suggests that early morning dopamine deficiency, frequently described in obese and T2D individuals, is involved in the overactivation of the sympathetic tone and release of corticotropin-stimulating hormone by the hypothalamic paraventricular nucleus. These effects produce substantial variability in daily heart rate, an indicator of cardiac autonomic neuropathy, and are associated with adverse events and dysmetabolic consequences on glucose control [64]. DR_2_ agonists may improve hemodynamics in T2D patients with heart failure (HF), positively affecting heart-failure-related outcomes [65]. Nevertheless, ergot-derived dopamine agonists are known for their cardiotoxicity due to their co-agonism with serotoninergic receptors [66]. Especially when administered at high doses, ergot-derived dopamine agonists are associated with myocardial valvopathy, thrombosis, arrhythmic events, and HF [66,67]. Antagonizing the serotoninergic effects of these agents may be considered a possible therapeutic strategy in diabetes-related HF [68]. From a therapeutic viewpoint, dopamine agents provide controversial evidence in terms of improvement in hemodynamics, preservation of renal function, and potassium homeostasis while on loop diuretics in advanced and acutely decompensated HF. Combining low-dose dopamine with low-dose loop diuretics effectively improves hemodynamic parameters and preserves glomerular filtration rate deterioration compared to high-dose loop diuretics alone [69]. Nevertheless, the results of two randomized clinical trials did not confirm the efficacy of low-dose dopamine in combination with both low-dose and high-dose diuretics in this clinical setting [70,71]. It is unclear if dopaminergic agents may be therapeutic in less severe clinical stages of HF to prevent adverse outcomes and reduce the risk of hospital admission due to symptomatic HF, but more investigation is ongoing (NCT01901809). It is unclear if positive results provided by SGLT2is on HF-related outcomes could depend, at least in part, on improved intracardiac dopamine metabolism.

Neurologic effects after ischemic stroke largely depend on the location and extension of ischemic areas, time of exposure to ischemic reperfusion injury, and baseline cerebral performance. Generally, ischemic stroke impairs dopamine release, synthesis, and DR activity in the striatum [72]. Dopamine deficiency is associated with cognitive and motor impairment, and evidence suggests that treatments restoring dopamine levels may improve recovery after stroke [73,74]. The mechanisms explaining this potential are that dopamine enhances motivation and improves symptoms of neuropsychiatric disorders related to stroke, complicating the rehabilitative period [75,76]. Nevertheless, no evidence has been provided to confirm the therapeutic rationale as a pharmacological strategy to improve relevant endpoints during post-stroke rehabilitation [77,78]. It is unknown if certain medications, such as thiazolidinediones, GLP-1RAs, and SGLT2is, may affect intracerebral dopamine metabolism as one of the mechanisms by which they benefit the prevention of ischemic stroke.

**Table 2:** Summary of evidence highlighting the role of dopamine in the pathogenesis of diabetes-related chronic complications and implication for therapy.

Diabetes-Related Traditional Chronic Complication	Role of Dopamine	Effect	Rationale for Treatment (Dopamine Agonists or Levodopa)
Retinopathy [45,46,47,48,49,50,51,52,53,54,55]	Impaired intraretinal metabolism (deficiency)	Defective photoreceptor adaptation to light	Yes
Chronic renal disease [56,57,58,59,60]	Impaired renal metabolism (glomerular filtration-depended reduction)	Dysregulation in water and natrium resorption; promotion of glomerular hyperfiltration; micro- and macroalbuminuria	Scanty evidence or negative results
Neuropathy [64,65,66]	Defective axonal transport; impaired metabolism (accumulation due to inadequate conversion to noradrenaline?)	Implication for painful neuropathy	No (dopamine antagonists)
Stroke [72,73,74,75,76,77,78]	Impaired cerebral metabolism (deficiency)	Loss of motivation, motor impairment, and pathogenic role in post-stroke neuropsychiatric disorder	Scanty evidence or negative results
Cardiovascular diseases [61,62,63,64,65,66,67,68,69,70]	Impaired cardiac metabolism (accumulation due to inadequate conversion to noradrenaline?); striatal deficiency	Increased risk of heart failure, impaired coronary vasodilatation, cardiac autonomic neuropathy	Scanty evidence or negative results

Table 2 summarizes the leading evidence indicating the role of dopamine in the pathophysiology of traditional diabetes-related chronic complications.

## 4. The Pathophysiological Link between Type 2 Diabetes and Chronic Disorders Characterized by Impaired Dopamine Activity/Metabolism

Dopamine plays a crucial role in the central nervous system (CNS), as it regulates many activities, including motor control, spatial memory, motivation, sleep, arousal, feeding, immune function, maternal behaviors, and lactation, to cite the main effects [79]. Dopamine signaling disruption is involved in the pathogenesis of several neurological and psychiatric disorders such as Parkinson’s (PD) and Huntington’s (HD) diseases, attention-deficit/hyperactivity disorder (ADHD), and addiction. Here, we discuss the potential and putative pathophysiological interconnection between T2D and these chronic neurological and psychiatric disorders characterized by impaired dopamine activity/metabolism (Table 3).

### 4.1. Parkinson’s Disease

PD is characterized by a primitive degeneration of a group of neurons in the substantia nigra and largely synapsing with basal ganglia. The distinctive neuropathological hallmarks are the Lewy bodies, α-synuclein aggregates, located in the substantia nigra with overtime spreading to neocortical and cortical regions (late-stage disease) [80]. Dopamine is the neurotransmitter of this intricate system, whose function is essential in planning motorial schemata, beginning and fine regulation of voluntary movements, postural control, and basal muscle tone [81]. Early clinical manifestations of PD include bradykinesia, postural and rest tremors, and muscle rigidity of limbs, neck, and trunk [82]. Nonmotor symptoms are also present. Some may be part of a prodromal syndrome, including depression and anxiety, sleep disorders, and constipation. Late-stage signs and symptoms of PD are related to relevant extrapyramidal imbalance, leading to motorial impairment and postural instability, accompanied by cognitive decline, manifesting in dementia and psychosis [82]. Regarding the frequency of clinical presentation, PD is the second cause of neurodegenerative diseases after Alzheimer’s dementia and affects 1 to 2 per 1000 of the general population [80]. Increasing age, genetic predisposition [83], exposure to pesticides and metals, and history of head trauma are the most common risk factors of PD [84]. T2D was found to increase the risk, accelerate the progression, and increase the severity of PD [85,86]. Diabetes severity, conceived as the number of anti-hyperglycemic agents used to treat hyperglycemia, long diabetes duration, need for insulin, and presence of chronic complications, is associated with an increased risk of developing PD [87]. Moreover, evidence suggests that diabetes affects nigrostriatal dopamine synthesis [88]. Therefore, similar pathways are involved in the pathogenesis of both diseases. The α-synuclein is largely involved in the pathogenesis of PD [89,90]. The protein is encoded by the SNCA gene located in chromosome 4; its native structure and function are still debated, but it is thought that α-synuclein is normally located in presynaptic terminals where it modulates neurotransmitter trafficking by affecting membrane plasticity [91]. Overexpression, posttranscriptional changes, aggregation of α-synuclein, and lysosomal dysfunction may facilitate the intracellular accumulation of the protein that is thought to induce membrane damage as the leading mechanism of neuronal injury [89,92,93]. Neuroinflammation, mitochondrial dysfunction, and impaired autophagy are other mechanisms of neurodegeneration in PD [94], and these mechanisms also characterize the pathophysiology of T2D [95,96]. Alpha-synuclein is essential in regulating insulin secretion at the level of β-cells [97] and glucose homeostasis in skeletal muscles, as demonstrated by experimental models [98]. The relationship between insulin and α-synuclein is interesting, as the latter can be considered a key regulator of insulin synthesis and activity in negative feedback [99]. In other words, the PI3K/Akt/GSK3β signaling could be involved in the pathogenesis of PD and Lewy body dementia as, in rodents, the overactivation of the pathway results in signs of neurodegeneration of the cortex and limbic system while silencing it ameliorates cognitive impairment [100]. Insulin and insulin-like growth factors act exactly by stimulating the phosphatidylinositol 3-kinase/serine-threonine protein kinase/glycogen synthase 3β (PI3K/Akt/GSK3β) pathway. It could be speculated that hyperinsulinemia, commonly found in prediabetes and early-stage T2D, may induce and accelerate neurodegeneration in the human brain [101]. On the other hand, impaired insulin signaling and insulin resistance are associated with the accumulation of α-synuclein (as well as β-amyloid, neurofibrillary tangles, and tau-proteins in Alzheimer’s disease) in PD [102]. Circulating insulin from β-cells can cross the blood–brain barrier and enter the CNS. In addition, insulin can be synthesized into the brain by several types of cells, such as astrocytes and neurons, in different cerebral areas, including the hippocampus, prefrontal cortex, dentate gyrus, thalamus, and olfactory bulb [103]. Insulin receptors are expressed in the same brain areas and VTA and substantia nigra, where insulin signaling regulates reward circuits modulating appetite and food intake [103]. Insulin exerts anti-inflammatory and anti-apoptotic properties, modulates mitochondrial function, improves autophagy and recycling of intracellular matter, and apoptosis in the brain [104]; therefore, as another hypothesis, intracerebral insulin resistance may contribute to neuroinflammation [105] and neurodegeneration [106]. The potential for neurotrophic protection of native GLP-1 is well known [107]. As observed for insulin, native circulating GLP-1 may enter the CNS, but it can also be synthesized in the brain, where several areas express GLP-1 receptors [108]. For example, native GLP-1 modulates appetite and food intake at the hypothalamic level [109,110]. Thanks to this mechanism, native GLP-1 is responsible for a sort of gut–brain talk, which was pivotal for the therapeutic application of GLP-1RAs to reduce appetite and promote weight loss [111]. Mechanistic studies found that GLP-1RAs may protect against neurodegeneration. In two rodent models of nigrostriatal injury, the administration of the GLP-1 agonist extendin-4, one week after the injury, arrested the progression of the nigrostriatal damage and restored the synthesis and release of dopamine [112]. Similarly, extendin-4 was found to promote neurogenesis and improve dopamine metabolism in the substantia nigra in an animal model of PD [113]. Years later, GLP-1RAs demonstrated neuroprotective effects in degenerative, ischemic, and traumatic models of brain injury [114]. Liraglutide provided evidence of reducing chronic neuroinflammation in response to X-ray irradiation [115]. Soon after, the neuroprotective effects of GLP-1RAs were also confirmed by neuroimaging in neurodegenerative diseases, including PD, in which treatment with GLP-1RAs provided a relevant increase in glucose uptake in specific cerebral areas as an indicator of improved metabolic activity [116]. The results of these studies posed the basis for further investigation into human disease [117]. Clinical trials have demonstrated that Exenatide, the most studied GLP-1RAs, improved motor and non-motor symptoms after one year of treatment in patients with PD [118,119,120]. More recently, other GLP-1RAs have shown promising preclinical protective effects on neurodegenerative diseases by providing synaptic protection, improvement in cognition, learning and motor function, amyloid pathology-ameliorating properties, improvement in intracellular calcium currents and endoplasmic stress, anti-inflammatory effects, reduction in oxidative stress, mitochondrial dysfunction and apoptosis, enhancements in the neuronal insulin sensitivity and energy metabolism, improvement in autophagy and mitophagy, and neurogenesis [121,122,123,124,125]. Overall, results for mechanistic and clinical trials indicate a tight relation between PD and T2D. Appropriate pharmacological management of T2D is expected to reinforce the dopamine imbalance in PD, also providing anti-oxidative and anti-neurodegenerative effects with overall improvement in PD-related prognosis.

### 4.2. Huntington’s Disease

Huntington’s disease (HD) is a neurodegenerative autosomal dominantly inherited disorder caused by a mutation of the Huntingtin gene located in chromosome 4. The mutation consists of a progressive (cross-generational) trinucleotide (CAG) triplet expansion, resulting in an extended polyglutamine sequence into the Huntingtin protein. When the number of CAG triplet repetitions is more than 35, the mutation makes missense changes in the native Huntingtin structure, compromising its functions, and the disease is most likely to occur [126]. Huntingtin protein is ubiquitously expressed, especially in the CNS, where it regulates the trafficking of vesicles and organelles, transcription, protein handling at the endoplasmic reticulum–Golgi level, and cell survival (anti-apoptotic activity) [127]. Clinical manifestations of HD occur in patients aged 30–50 years and are characterized by motor symptoms consisting of involuntary movements (chorea), memory loss, progressive cognitive decline, and psychiatric disorders [128]. HD evolves through several degrees of muscular and cognitive impairment. Late-stage disease is characterized by severe motor impairment resulting in bradykinesia, akinesia, dysarthria, and dysphagia with relevant deterioration of residual functional capabilities [129]. From a pathophysiological viewpoint, HD is defined by a degeneration of medium-sized spiny neurons of the striatum, with a marked impairment of γ-aminobutyric acid (GABA)-ergic signaling, followed by a progressive retrograde degeneration of cortical pyramidal neurons (which project to the striatum) and neuroanatomical disconnection between the striatum and the substantia nigra [130]. Mechanistic studies found significant neurochemical changes in HD, such as a decreased concentration of the inhibitory neurotransmitter GABA, mostly responsible for the extrapyramidal symptoms of the disease, associated with increased concentrations of dopamine and serotonin in the basal ganglia [131]. Across the stages of the disease, a progressive reduction in cannabinoid, dopamine, and adenosine receptors has been described in the basal ganglia [132]. Studies conducted with animal models demonstrated that the early phase of HD could be characterized by a relevant dopamine deficiency, hence suggesting that nigrostriatal degeneration could be one of the first pathophysiological events of the disease [133]. However, the hyperkinetic manifestation, defining the most common clinical presentation of HD in human pathology, suggests the contrary with regards to dopamine metabolism, so that striatal dopamine excess may have a role in the pathophysiology of HD [134]. The evidence of monoamine-oxidase enzymatic hyperactivity in the basal ganglia could indicate aberrant dopamine metabolism at that level, thus confirming the hypothesis. Excessive dopamine catabolism by monoamine oxidase produces a relevant amount of hydrogen peroxide, which, in turn, contributes to oxidative stress, a well-recognized pattern of damage in HD [135]. Mitochondrial dysfunction, impaired energy metabolism, axonal transport, microglial inflammation, and decreased synthesis of brain-derived neurotrophic factors are the other key factors of HD pathophysiology [136,137]. Beyond the fact that HD is a genetic disorder, it is known that patients with HD exhibit a greater frequency of prediabetes and diabetes than the general population [138,139]. The origin of impaired glucose metabolism in HD is difficult to understand. First, it was demonstrated that age-related decline in β-cell number was more evident and rapid in the HD model than in controls; nevertheless, this phenomenon was not associated with developing insulin deficiency, and most animals survived without developing diabetes lifelong. However, other experiments indicated that Huntingtin mutation was associated with impaired insulin synthesis and vesicular transport, suggesting that hyperglycemia was attributable to insulin deficiency. Conversely, HD models were prone to accumulate weight and expand visceral adipose tissue due to more food intake and hypothalamic dysfunction. As an additional mechanism, the onset of motor symptoms was associated with relevant impairment of cerebral glucose consumption, which could result in less overall glucose utilization and more glucose excretion. The hypothesis was confirmed by the evidence of a significant increase in glycosuria, but diabetes occurred only in a few rats, suggesting that the mechanism was insufficient to explain impaired glucose metabolism. Concurrent and disease-related chronic stress was considered a possible mechanism underlying chronic hyperglycemia, as well as mitochondrial dysfunction and inflammation [140,141,142]. One recent study elucidated the potential mechanism explaining glucose impairment in HD using mouse pancreatic insulinoma cells (line NIT-1) expressing N-terminal mutant Huntingtin containing 160 polyglutamine. More precisely, it was found that insulin receptor substrate 2 (IRS-2) expression decreased, and the remaining was recruited into mutant Huntingtin aggregates [143]. IRS-2 is essential for activating PI3K/AKT/FoxO1 to mediate glucose stimulation into insulin secretion. As an additional mechanism, exogen insulin administration inhibited the formation of mutant Huntingtin aggregates, thus removing the block on the PI3K/AKT/FoxO1 pathway and ameliorating pancreatic insulin reserve [143]. Conversely, T2D is associated with earlier onset and faster progression of HD [144]. The basic mechanism of this relationship involves the impairment of the PI3K/Akt/mTOR pathway described in T2D and HD [145]. The pathway regulates various functions, including cell proliferation, survival, apoptosis, autophagy, protein synthesis, glucose metabolism, angiogenesis, cytoskeletal organization, and vesicular trafficking [146]. Hyperglycemia leads to hyperinsulinemia, insulin resistance, and the formation of advanced glycation end products (AGEs), which interact with a specific receptor (RAGE). The interaction between AGEs and RAGE stimulates different intracellular pathways involved in diabetes-related tissue damage, such as mitochondrial and endoplasmic stress, oxidative stress, intracellular protein aggregation, and inflammation. In addition, AGEs downregulate the PI3K/Akt/mTOR pathway, further impairing cellular functions, as mentioned above, and deterioration of glucose control [147]. This mechanism, which could be defined as a vicious circle, may explain why T2D may deteriorate the prognosis of HD [148,149]. Besides the effect on endogenous dopamine and the above considerations for PD, optimal glucose control would result in better outcomes in HD. Even though no specific clinical trials have been conducted so far, AMP-activated protein kinase AMPK activation (by metformin) has been demonstrated to improve motor and cognitive outcomes in experimental models of HD [150,151,152]. Targeting insulin signaling for restoring insulin sensibility may have a therapeutic rationale in stimulating glial cells to produce neurotrophic factors, attenuating mutant huntingtin precipitates, reducing neuroinflammation and neurotoxicity, and improving autophagy [153,154,155,156,157].

### 4.3. Attention-Deficit/Hyperactivity Disorder

Attention-deficit/hyperactivity disorder (ADHD) is a neurodevelopmental disorder defined by impairing levels of inattention, disorganization, and/or hyperactivity–impulsivity [158]. The estimated prevalence of ADHD is around 6–8%, and it is usually diagnosed in childhood or young adults more in boys than girls [159]. A variable number of gene mutations could be involved in the pathogenesis of ADHD, including those targeting dopaminergic and serotoninergic pathways [160]. It is thought that ADHD, one of the most common inherited mutations, is biologically attributable to impaired dopaminergic signaling rather than impaired dopamine synthesis. Most common polymorphisms of dopamine receptors, such as DR2, have been described, involved in reward to environmental factors, DR_4_ and DR_5_ [161]. Real-world data revealed that ADHD is a risk factor for T2D and related comorbidities such as overweight and obesity [162], and it is associated with poor glucose control in young individuals with type 1 diabetes [163]. The results of a meta-analysis confirmed that the adjusted risk of T2D is more than doubled in patients with ADHD than in those without [164]. In addition, ADHD is more frequently diagnosed in individuals born by mothers who developed diabetes during pregnancy, suggesting that maternal hyperglycemia may be an epigenetic risk factor for ADHD [165]. Risks seem to be related to some specific adverse neonatal outcomes such as very and extremely preterm and very and extremely low birth weight as well [166]. The importance of such epidemiological data has been recognized by guidelines recommending implementing medical management of T2D in people living with ADHD [167]. Compulsive eating behaviors and binge eating disorders are frequently associated with ADHD, especially in young patients exposed to a higher risk of weight gain over time [168,169], and complicate glucose management and control of body weight in T2D. Pharmacological management of eating disorders in ADHD is therefore recommended. Most approved medications for ADHD, such as methylphenidate, seem to improve several aspects of behaviors more than placebo or non-pharmacological interventions (e.g., cognitive training and psychotherapy) [170]. Targeting dopaminergic (and noradrenergic) reuptake or stimulating dopamine synthesis represents the rationale of pharmacological intervention in ADHD. Reinforcing dopaminergic pathways in ADHD and obese individuals enhances the reward value of food [171] with positive consequences on food intake, caloric restriction [172], and body weight control [173,174]. Nevertheless, both methylphenidate and amphetamine reduce insulin secretion in a dose-dependent manner. Therefore, it is expected that high-dose treatment may potentially impair glucose control, requiring more appropriate glucose management [175]. So far, no specific clinical trials have been conducted in this cluster of patients, and the management of hyperglycemia is essentially the same in the general population. However, a tight neuroanatomical relation between dopaminergic circuits and GLP-1 sites of action in the brain, such as the amygdala, hypothalamus, hippocampus, and NAc, is known and should not be overlooked [176]. These areas regulate the most relevant cerebral function, including memory, food intake, and motivation. Potentiating dopaminergic pathways, especially DR_2_ agonism, which is usually impaired in ADHD as well as obesity, by GLP-1RAs would result in caloric restriction and weight loss [177,178]. Besides the controversy on the likelihood that GLP-1RAs act at several brain districts, exactly as observed for native GLP-1, specific trials are needed to clarify if this class of medication has feasible therapeutic opportunities in ADHD patients.

### 4.4. Addictions

Addiction refers to a psychopathological condition in which one individual is unable to control a specific impulse to carry out reiterating actions or behaviors that hesitate in physical or psychological dependence on something [179]. In pharmacology, addiction is defined as a chronic and relapsing disorder in which individuals are prone to and become dependent on compulsive seeking of abuse substances or drugs for achieving pleasure or feeling better [180]. Addiction is the leading cause of dependence on specific behaviors [181] or chemicals, such as alcohol [182], nicotine [183], certain medications (e.g., opiates) [184], and illicit substances [185]. The underlying mechanisms of addiction are complex and involve several brain areas of the reward circuit and dopaminergic (mesolimbic/mesocortical) pathways [186]. While nigro-striatal dopaminergic pathways are involved in regulating feeding [187] and impaired dopamine activity is associated with reduced food intake at this level, mesolimbic dopaminergic pathways seem to be involved in other aspects of food intake such as motivation, reward, and hedonic food [188]. Food and eating addiction [189], carbohydrates, and fat addiction are considered neurobehavioral, psychopathological, and maladaptive dysfunctions associated with exaggerated caloric intake, obesity, and possibly eating disorders, including binge eating [190]. Recent research confirms that the same mechanisms underlying dependence on drugs and substances of abuse are involved in food addiction [191], with dopaminergic pathways and reward circuitry playing a crucial role [191]. Importantly, food intake is also associated with gratifying stimuli from the limbic system in a way that can be neurobiologically translated into an increase in mesolimbic and mesocortical dopamine levels and activation of reward circuits, as observed in alcohol dependence [192]. Although food addiction is not currently classified as an eating disorder or an independent condition, it is more frequently observed among overweight and obese patients [193]. Moreover, being far from describing a well-established pathophysiological link between food addiction and hormonal parameters, some interesting glucometabolic, inflammatory, and neurohormonal biomarkers could relate to this condition [194]. Overeating is associated with specific neurochemical and neurobiological changes in the CNS, mostly attributable to an imbalance between homeostatic, cognitive, and hedonic homeostasis. Neuroimaging studies found that obese compared to lean people had reduced mesolimbic and mesocortical expression of DR_2_ and reduced neuronal metabolic activity in these areas, leading to an impaired reward system [195]. Conversely, obese individuals have a greater baseline metabolic activity in the somatosensitive cortex representing the mouth, lips, and tongue, in other words, the leading cerebral region involved in the conscious processing of food palatability [196]. Low and very low calorie diets, selectively restrictive diets, diets plus behavioral interventions, or physical exercise are the most effective non-pharmacological treatments to induce weight loss and improve body composition in patients with obesity [197,198]. Despite optimal results in the short term, only a high adherence to diet recommendations and calorie restrictions may provide long-term benefits to prevent weight gain or weight regain after dietary-induced weight loss [199]. No specific diet protocols have been shown to ensure weight maintenance over time (usually no more than 6 months) [200,201], so weight regain is frequently observed in patients who discontinued calorie restriction or those not receiving adequate food education or behavioral intervention [202]. The causes of this common but difficult-to-manage phenomenon include several mechanisms that can be considered as an adaptation to weight loss following calorie restriction. The drastic decline of leptin levels and shortage of circulating free fatty acids, both associated with diet-induced adipose tissue shrinkage, are the peripheral mechanisms of weight regain after diets. Inflammation, metabolic adaptation to calorie restriction, metabolic shift toward carbohydrate utilization upon their reintroduction (i.e., after ketogenic diets), and neuroendocrine adaptation to weight loss are other common mechanisms of weight regain [203,204,205]. Most importantly, diet and a healthy lifestyle cannot reverse brain attraction to food intake, and this effect may be considered as another important mechanism of weight regain after diet discontinuation [206]. The VTA is crucial in perceiving rewarding environmental stimuli and activating specific behaviors to obtain future rewards. Function crosstalk in the mesolimbic system between the VTA, NAc, hippocampus, and AMY is necessary to carry out the functions mentioned above, and dopamine is the key neurotransmitter. Food intake directly stimulates VTA activation by an intricate series of neurotransmitters and neuromodulators that include opioids, GABA, glutamate, and acetylcholine [207]. After the activation, neurons in the VTA fire to dopaminergic neurons in the NAc, resulting in dopamine synthesis and the release and activation of reward circuits in response to food intake [207]. Most importantly, orexigenic hormones affect dopaminergic activity at that level, being involved in food reward and hedonic eating [208]. Ghrelin, a stomach-derived polypeptide, is secreted in response to calorie restriction. Ghrelin receptors have been described in the VTA, where the hormone exerts a bimodal effect on dopamine synthesis and release. In the presence of normal food intake or consumption of palatable food, ghrelin was found to stimulate the mentioned dopaminergic pathway, hence motivating reward and gratification after meal ingestion [209,210]. This effect is thought to be mediated by mu and kappa opioid receptors in the VTA [211] and neuropeptide Y [212]. On the other side, ghrelin stimulation without food intake attenuates dopamine release. Other orexigenic hormones may modulate dopamine release in the VTA, as observed for ghrelin, while anorexigenic hormones may act in the opposite way [213]. Interesting experiments in rodents have found that GLP-1RAs may also affect dopaminergic pathways involved in addictions. Intracerebral administration of exendin 4 in the NAc, but not in the VTA, significantly attenuated alcohol-induced locomotor stimulation and memory of alcohol reward, as well as decreased alcohol intake [214]. Similarly, peripheral and central administration of exendin 4 attenuated cocaine-induced locomotion and abuse by blunting dopamine release in the VTA and NAc in response to cocaine administration [215]. Despite some controversial results, GLP-1RAs may reduce dopamine release by the mesolimbic/mesocortical pathways in response to food intake [216,217]. In other words, besides a direct effect on appetite, GLP-1RAs may reduce meal-induced gratification after food intake, foster lower caloric intake, and promote weight loss. Targeting dopamine metabolism also positively affects energy balance and weight loss, as observed with specific dopaminergic/noradrenergic reuptake inhibitors [218,219,220]. Overall, GLP-1RAs have the potential to modulate dopamine metabolism in addictions by contrasting positive rewards related to chronic and repetitive exposure to novice stimuli, such as pathological food intake or exposure to substances of abuse.

**Table 3:** Pathophysiological mechanisms linking type 2 diabetes and chronic disorder characterized by impaired dopamine activity/metabolism.

Diseases and Conditions	Pathophysiological Mechanisms
Diabetes and Parkinson’s disease [85,86,87,88,89,90,91,92,93,94,95,96,97,98,99,100,101,102,103,104,105,106,107,108,109,110,111,112,113,114,115,116,117,118,119,120,121,122,123,124,125]	Impaired striatal dopamine synthesis Oxidative stress Insulin resistance (cerebral and systemic insulin resistance) Inflammation (neuroinflammation and systematic inflammation) Aberrant α-synuclein metabolism Mitochondrial dysfunction Impaired autophagy and apoptosis
Diabetes and Huntington’s disease [135,136,137,138,139,140,141,142,143,144,145,146,147,148,149,150,151,152,153,154,155,156,157]	Exaggerated dopamine catabolism Mitochondrial dysfunction Impaired energy metabolism and axonal transport Neuroinflammation and reduced synthesis of brain-derived neurotrophic factors Impaired insulin synthesis and release (PI3K/AKT/FoxO1-IRS2 pathway) Impaired cell proliferation, survival, apoptosis, autophagy, protein synthesis, glucose metabolism, angiogenesis, cytoskeletal organization, and vesicular trafficking (PI3K/Akt/mTOR pathway)
Diabetes and ADHD [161,162,163,164,165,166,167,168,169,170,171,172,173,174,175,176,177,178]	Impaired dopamine synthesis Behavioral and disordered eating
Diabetes and addictions [191,192,193,194,195,196,197,198,199,200,201,202,203,204,205,206,207,208,209,210,211,212,213,214,215,216,217,218,219,220]	Impaired dopamine synthesis (reward and hedonic food) Disordered eating or eating disorders Obesity-related concerns

Table 3 summarizes the mechanisms linking the pathophysiology of T2D and the most relevant diseases in which dopamine activity/metabolism is impaired. Insulin resistance, mitochondrial dysfunction, neuroinflammation, impaired autophagy, and impaired neurogenesis are the most common mechanisms linking T2D and neurogenerative diseases (such as Parkinson’s and Huntington’s diseases). In ADHD and addictions, behavioral disorders inducing eating disorders are crucial elements that expose affected patients to T2D and related complications. Improving dopaminergic pathways results in improved clinical outcomes.

## 5. Therapeutic Implications

Here, we discuss the pharmacological role of dopamine in T2D and of anti-hyperglycemic medications in T2D and concomitant diseases in which dopamine activity/metabolism is impaired to overview existing and lacking evidence indicating that specific drugs may improve the pathophysiology and clinical manifestation of the above-mentioned disturbances. Most evidence refers to PD due to its more epidemiological relevance than other dopamine-related conditions. However, more information about the potential effects of approved treatments for T2D and chronic neurologic and psychiatric disorders characterized by impaired dopamine activity/metabolism on specific disease-related outcomes has been summarized in Table 4.

The intrapancreatic uptake of levodopa increases after glucose exposure, and hyperglycemia may sustain the machinery of dopamine synthesis in β-cells. Animal models showed that dopamine directly suppresses insulin secretion by curbing the duration of the action potential in response to β-cell glucose entry. The leading effect is mediated by the D_2_ receptor signaling [221]. In an experimental model, D_2_ overexpression abolished the glucose-stimulated Ca2+ influx and insulin secretion in β-cells. This toxic effect was partially reverted after the treatment with a D_1_-D_2_ heterodimer agonist (D_1_ and D_2_ dimerized on β-cell surface), suggesting that the D_1_ receptor may protect β-cells from the harmful effects of dopamine by modulating D_2_ signaling [222]. Given the role of D_2_ receptors in mediating the intrapancreatic effects of dopamine [223], specific treatment could be necessary to prevent or improve metabolic disorders associated with neurological and psychiatric conditions and their related treatment [224,225].

Bromocriptine, a DR_2_ agonist, is an FDA-approved oral medication for T2D, available in the US as tablets of 0.8 mg. The maximal approved daily dose is 4.8 mg, corresponding to 8 tables per day [226]. The tables are administered at fasting as food intake significantly affects the gastric adsorption of bromocriptine. Bromocriptine peaks 60 min after the table assumption, with a half-life of around 6 h, and undergoes relevant first-pass hepatic extraction and clearance so that only less than 10% of the assumed dose has a therapeutical effect [227]. Bromocriptine seems to replace the reduced dopaminergic tone in the hypothalamus, observed in insulin-resistant, obese, and diabetic patients, and reduce the serotoninergic tone, which is responsible for increased appetite and preference for carbohydrate-rich and hypercaloric foods [226]. Thanks to this mechanism, bromocriptine reduces the appetite and improves peripheral insulin sensitivity. In addition, bromocriptine reduces insulin and glucagon secretion by acting directly on DR_2_ on β- and α-cells, respectively [228]. Bromocriptine produces a 0.6–0.7% reduction in HbA1c levels in T2D when assumed alone (monotherapy) or in variable combinations with other anti-hyperglycemic agents also providing cardiovascular benefits [229]. Dopamine agonists, including bromocriptine, have been approved for PD [230]. Dopamine agonists effectively improve motor symptoms, delay levodopa replacement, and reduce fluctuation in dopamine levels over time [231]. The benefit of dopamine agonists could depend, at least in part, on low oxidative stress due to replacing dopamine deficiency instead of stimulating the neurotransmitter synthesis by L-DOPA supplementation [232,233]. However, the long-term efficacy of bromocriptine is lower than that of L-DOPA, and treatment with dopamine agonists is usually weighted down by adverse events such as mitral valve damage, impulse control disorders, and compulsive behaviors [234,235]. Other typical adverse events are headache, dizziness, hypersomnia, dyskinesia, psychosis, hypotension, tachycardia, nausea, and nasal congestion. Overall, dopamine agonists may be preferred as early-stage treatment of PD and could be considered a treatment option to improve glucose control in patients with both conditions (PD and T2D).

An experimental study indicated that the expression of molecules involved in DR_2_ signaling is increased in islets from high-fat-diet obese mice. The whole grain-derived γ-oryzanol, a mixture of vegetal sterols, improved glucose control, promoted weight loss, and reduced appetite and caloric intake by acting at the hypothalamic level [236]. Oryzanol is found in rice and bran oils, tomatoes, and green peas. Given its lipophilic nature, γ-oryzanol crosses the blood–brain barrier and enters the CNS, where it provides anti-oxidative and anti-inflammatory properties and reduces the endoplasmic reticulum stress (an indicator of relevant cell damage) [237]. With a similar mechanism, γ-oryzanol may protect against β-cell damage [238]. Moreover, γ-oryzanol enhances glucose-dependent insulin secretion by activating the protein kinase A pathway. This mechanism is thought to suppress the dopamine signaling, thus providing a beneficial effect on insulin secretion and β-cell survival [239]. Preclinical studies confirmed that γ-oryzanol may reduce neuromotor deficits, dopamine depletion, and oxidative stress in models of PD [240,241]. Overall, γ-oryzanol may positively affect T2D by stimulating insulin secretion and neurodegenerative disorders due to anti-oxidative and anti-inflammatory effects. However, it may hamper dopamine signaling. The magnitude of this effect is unclear in specific conditions such as PD and HD and should be elucidated.

Metformin is a synthetic biguanide approved as first-line treatment in T2D due to several effects, including mild suppression of gluconeogenesis in the liver, reduction in glucose absorption in the gut, enhancement of glucose utilization by mitochondria in peripheral tissues, and induction of weight loss [242]. Most of the therapeutic effects of metformin are mediated by the activation of AMPK, which is impaired in T2D and chronic neurodegenerative diseases, including PD [243]. Metformin-mediated AMPK activation can result in potentially favorable effects in PD as it regulates cellular energy metabolism, autophagy, mitochondrial performance, redox homeostasis, and anti-inflammation [244,245,246]. The branched-chain amino acid transferase (BCAT-1) is a critical regulator enzyme involved in the metabolism of leucine, isoleucine, and valine to transmute them into terminal products of the mitochondrial oxidative chain such as acetoacetate and succinyl-CoA [247]. Early-stage PD is characterized by mitochondrial hyperactivity, excessive oxidative stress, and production of oxygen-reactive species, and BCAT-1 has a role in mediating mitochondrial-hyperactivity-induced neurotoxicity. As metformin attenuates mitochondrial hyperactivity, it could have a therapeutic role in early-stage PD [248]. The activation of AMPK in the brain decreases the expression of β-secretase 1 protein, which plays a crucial role in producing cleavage products such as β-amyloid and α-synuclein [249]. Moreover, metformin may increase the level of acetylcholine at the presynaptic site by suppressing the synthesis and activity of the acetylcholinesterase and, therefore, may play a crucial role in maintaining memory function [250]. Besides the positive results of preclinical mechanistic studies, a few data have been published in humans with controversial data on the efficacy and effectiveness of metformin in PD. One real-life study found that low-dose but not high-dose exposure to metformin (considering 2 g of metformin as the defined daily dose) reduced the odds of PD in T2D [251]. Hence, one could speculate that low-dose metformin may be sufficient to provide positive effects, as described above, while exposure to high-dose metformin may result in potential toxicity. The actual mechanism of this “metformin-related” neurotoxicity could be attributable to some secondary (and dose-dependent) effects of chronic treatment. One of these is the reduction in circulating levels of Vitamin B12, as metformin was found to impair its absorption at the intestinal level in a dose-dependent manner [252]. Vitamin B12 deficiency has been found to affect cognition and may play a role in cognitive impairment and motorial deterioration [253]. Being far from confirming this hypothesis, baseline assessment of Vitamin B12 and periodic monitoring of its circulating levels during the follow-up could be reasonable in patients with T2D on metformin, especially those with or at risk of cognitive impairment or neurodegenerative disorders, including PD. Other common side effects of metformin include gastrointestinal discomfort, nausea, diarrhea, and abdominal pain. Adverse effects of metformin are usually mild and self-limiting, and specific advice usually reduces their incidence and severity. Metformin should be prescribed at low doses (e.g., 500 mg once daily) with gradual titration over time (usually a few days or weeks). Moreover, the post-meal assumption of metformin usually attenuates gastrointestinal discomfort. In case of persisting or recurring adverse events, switching to long-release formulations is recommended. Overall, more trials are needed to understand better and elucidate the therapeutic role of metformin in patients with PD (e.g., NCT05781711).

Acarbose, an α-glucosidase inhibitor, attenuates the intestinal absorption of carbohydrates. Thanks to this mechanism, acarbose improves glucose control, especially by reducing post-prandial glucose amplitude and insulin spikes [254]. In addition, acarbose may have a role in suppressing the synthesis of proinflammatory cytokines by increasing a key regulator microRNA (10a-5p) in the ileum that is overexpressed in T2D and PD [255]. These effects are probably insufficient to explain the potential rationale of acarbose in treating T2D patients with PD. To our knowledge, no specific studies have been published, nor are there ongoing trials to assess the efficacy/effectiveness of acarbose in this cluster of patients.

Apart from the anti-hyperglycemic properties, peroxisome-proliferator-activated receptor (PPAR) agonists have shown promising neuroprotective effects. In T2D, the foremost PPAR-γ agonist, pioglitazone, has relevant anti-inflammatory and anti-atherosclerotic properties [256]. Several mechanisms explain the anti-atherosclerotic effects of PPAR-γ agonists, inducing a potent insulin-sensitizing action that improves glucose uptake and metabolism by peripheral tissues (skeletal muscle and adipose tissue); a potent modulating effect on lipid metabolism by reducing circulating levels of free fatty acids and low-density lipoprotein and increasing high-density lipoprotein; and relevant modulation of the cytokine signature of adipose tissue by simulating adipocyte to release Adiponectin and Leptin and suppressing the release of resistin [257]. In addition, pioglitazone has been demonstrated to reduce neuroinflammation after cerebral injuries (i.e., ischemia, brain trauma) [258]. Preclinical trials showed that pioglitazone administered subcutaneously during the reperfusion phase in a rat model of experimental cerebral ischemia provided a relevant reduction in cerebral infarction by around 30% [259]. Thanks to these effects, pioglitazone has gained much interest in preventing or treating cerebrovascular diseases in T2D [260]. Clinical data showed that pioglitazone was associated with a reduced risk of cerebrovascular events by 38% and 24% in two secondary prevention trials, respectively [261,262]. Other trials confirmed the cardio and cerebrovascular protection of pioglitazone in T2D patients [263]. The rationale of pioglitazone as a background treatment in chronic cerebrovascular diseases has also been considered for other conditions, including degenerative and traumatic cerebral diseases [264,265] and cognitive impairment [266]. Despite the solid rationale [267,268,269,270], the level of evidence is limited, and the results of clinical trials are still inconclusive or debated [271,272]. In one meta-analysis of clinical studies [273], pioglitazone reduced the risk of PD in patients with T2D [274,275]. Moreover, a Finnish nationwide case-control study found that, among the anti-hyperglycemic medications, only pioglitazone decreased the risk of incident PD in T2D [276]. Whether or not the results of these studies may be translated into real benefit in PD irrespective of T2D and how pioglitazone may modify the progression of PD in the early stage of the disease are unclear [277].

Dipeptidyl peptidase IV inhibitors (DPP-IVis) are a class of oral gliptins approved for T2D. These agents compete with endogenous incretins at the catalytic site of the enzyme and delay the incretin degradation [278]. Therefore, DPP-IVis improve glucose control by extending the circulating half-life of endogenous incretins, especially in the post-prandial phase, with satisfactory efficacy and durability [279,280]. Mechanistic preclinical studies using animal models of PD found that DPP-IVis had the rationale for targeting some specific mechanisms involved in the pathophysiology of motor degeneration as observed for Vildagliptin (suppressed the nuclear factor-kB and normalized the expression of the RAGE) [281,282], Sitagliptin (anti-inflammatory and anti-apoptotic properties; enhanced the expression of brain-derived neurotrophic factor) [283,284], Linagliptin (increased the levels of superoxide dismutase, catalase, and glutathione; elevated the levels of striatal dopamine; reduced the levels of proinflammatory cytokines) [285,286], and, with some controversial results, Saxagliptin [287,288]. Translating preclinic data into clinical potential is challenging. No specific clinical trials have been conducted, but data from real-life studies reported a relevant reduction in PD risk in patients with T2D on any DPP-IVis [289,290]. A relevant revitalizing effect of DPP-IVis against aging-related nigrostriatal deterioration has also been reported [291]. These positive effects may result from DPP-IVis capability to enhance dopamine synthesis in the basal ganglia [292]. However, controversial results were also published, and a recent meta-analysis did not confirm the therapeutical potential of DPP-IVis [293], over other agents, such as pioglitazone [276].

GLP-1RAs are promising agents. As mentioned above, Exenatide provided reliable evidence of improving motor and non-motor symptoms in PD, but its role in reducing the pathophysiology and background brain injury in humans currently needs to be clarified [294]. Promising results have also been elucidated by a preclinical study with Dulaglutide, in which GLP-1RAs were demonstrated to reduce neuroinflammation and promote neurodegeneration [295]. However, more investigation is essential to clarify several issues, including the potential for preventing the onset of degenerative diseases, including PD, in predisposing clinical conditions, such as T2D, efficacy and safety, and durability of treatment in the early and late-stage disease. With these purposes, ongoing trials are investigating the role of GLP-1RA analogs in PD; these include Liraglutide (NCT02953665), Semaglutide (NCT03659682), Exenatide again (NCT04232969, 48-week study; NCT04154072-NCT04305002, early-stage PD), and Lixisenatide (NCT03439943). It should be considered that GLP-1RAs induce relevant weight loss and reduce appetite, thus limiting their use in patients with low-weight-related concerns, undernourishment, or reduced motivation for food intake. GLP-1RA-associated adverse events are usually mild-to-moderate and self-limiting over a few weeks of treatment. Additionally, concomitant drugs (e.g., metformin) may increase the risk and severity of gastrointestinal adverse events. Adequate titration of GLP-1RAs is essential to minimize the risk of adverse events and to manage better side effects once they have occurred. A lower effective dose should be prescribed in sensitive patients, and in the case of persisting or relapsing adverse events, the switch to other GLP-1RAs or other classes of drugs is recommended. The potential for GLP-1RA-related retinal injury should also be considered.

More recently, the glucose-dependent insulinotropic peptide (GIP) has gained endorsement as a promising therapy in T2D. Dual agonists GIP/GLP-1RA, rather than single GIP agonists, have been shown to improve glucose control and induce relevant weight loss significantly better than previously observed with GLP-1RAs alone [296,297,298]. Moreover, promising results have been obtained by experimental models in mice where dual agonists compared to single GLP-1RAs attenuated more significantly relevant signs of neuroinflammation and neurodegeneration (such as α-synuclein) and boosted regenerative stimuli [299,300,301]. It is expected that dual GIP/GLP-1RA agonists may have a therapeutic role in such neurodegenerative disorders [302,303], and specific clinical trials are urgently needed.

SGLT2is impairs glucose resorption at the level of the proximal tubule in the kidney [304]. Thanks to this mechanism, SGLT2is induce a relevant reduction in renal glucose resorption threshold with a relevant increase in the rate of daily renal glucose excretion (glycosuria) [305], exerting an insulin-independent anti-hyperglycemic effect [306,307]. SGLT2is and GLP-1RAs [308] provided evidence to improve relevant extra-glycemic outcomes in T2D, especially at the cardiovascular and renal levels [309]. SGLT2is is demonstrated to affect glucose metabolism positively, provide more efficient energy utilization at the cellular site, and reduce oxidative stress and inflammation, which could be considered relevant targets for treating neurodegenerative diseases [310]. Dapagliflozin (1 mg/kg/day, for 3 weeks) improved motor coordination, diminished the expression of α-synuclein and related pathological alterations, augmented the level of expression of tyrosine hydroxylase and, consequently, dopamine in the basal ganglia, and reduced oxidative stress and neuroinflammation (by decreasing the activity of the NF-κB pathway and TNF-α levels) of a PD rodent model [311]. Similar findings were obtained with Empagliflozin [312], which also reduced endoplasmic reticulum stress and improved autophagy in rodents [313,314]. More recently, a computational study found that SGLT2is (Canagliflozin and Empagliflozin) displayed binding affinity and stability to the distal ubiquitin-binding domain, serving as possible inhibitors of the ubiquitin-specific protease 30 or USP30 that plays a crucial role in the pathogenesis of mitochondrial dysfunction in PD [315]. Real-world data show that SGLT2is can work to prevent neurodegenerative diseases in T2D [316], but available data did not include specific studies assessing the potential benefit of this class of medications on neurological outcomes [317].

**Table 4:** Summary of potential effects of approved treatments for type 2 diabetes and chronic disorders characterized by impaired dopamine activity/metabolism on specific disease-related outcomes. The present list includes the most common approved medication summarized by specific therapeutic areas and conditions for which the drug has been approved.

List of Medications	Therapeutic Area	Approved for	Mechanism of Action	Beneficial Effects *	Detrimental Effects *
Metformin	Diabetology	Diabetes mellitus	AMPK activator	Enhancement of cellular energy metabolism, improvement of autophagy and mitochondrial performance and redox homeostasis, anti-inflammatory effect, reduction in β-secretase 1 expression (AMPK activation) [244,245,246,247,248,249,250]	Dose-dependent adverse effects (abdominal pain or discomfort, nausea, diarrhea), impaired intestinal adsorption of Vitamin B12 [251,252,253]
Acarbose	Diabetology	Diabetes mellitus	Intestine α-glucosidase inhibitor	Reduction in synthesis of biomarkers associated with adverse outcomes (proinflammatory cytokines, microRNA 10a-5p) [254,255]	Adverse intestinal effects
Pioglitazone	Diabetology	Diabetes mellitus	PPAR-γ agonism	Anti-inflammatory and anti-atherosclerotic properties, insulin-sensitizing effect, attenuation of neuroinflammation [264,265,266,267,268,269,270,271,272,273,274]	Weight gain, water and sodium retention, intensive monitoring, or contraindication in case of heart failure, renal insufficiency, and macular edema
Gliptins	Diabetology	Diabetes mellitus	DPP-IV inhibitors	Suppression of NFkB, reduction in the expression of RAGE, anti-inflammatory and anti-apoptotic properties, enhancement of brain-derived neurotrophic factors, reinforcement of anti-oxidative systems, increase in striatal dopamine synthesis [281,282,283,284,285,286,287,288,289,290,291,292]	-
GLP-1RAs	Diabetology	Diabetes mellitus	GLP-1 agonism	Contrasting nigrostriatal injury and promoting neurogenesis, improvement of neuroinflammation and neuronal metabolic activity [118,119,120,121,122,123,124,125]	Relevant weight loss and reduction in appetite, potential for retinal injury
Gliflozins	Diabetology	Diabetes mellitus	SGLT2 inhibitors	Improvement of energy utilization, reduction in oxidative stress and neuroinflammation, improvement of endoplasmic stress and autophagy, potential for exogen USP30 inhibitor [310,311,312,313,314,315,316,317]	Potential risk of genitourinary infections, hypotension, dehydration, and rapid decline of renal function, especially in older patients
Secretagogues (Sulphonylureas, Glinides)	Diabetology	Diabetes mellitus	K inward channel inhibitors	Relevant improvement in short-term glucose control [318]	High risk of hypoglycemia, short durability, lack of evidence of extra-glycemic benefits, increased risk of dementia [319]
Cabergoline, Bromocriptine, Apomorphine, Pramipexole, Rotigotine	Endocrinology/ Neurology	Diabetes mellitus Parkinson’s disease	Dopamine agonism	Improved glucose control; improved motor symptoms; reduced oxidative stress; possible CV benefits [226,227,228,229,230,231,232,233,234,235]	Mitral valve damage; impulse control disorders; short-term efficacy
Entacapone, Tolcapone, Opicapone	Neurology	Parkinson’s disease	COMT inhibitors	Not well established	-
Rivastigmine	Neurology	Parkinson’s disease	Acetylcholinesterase inhibitor	Not well established	-
Amantadine	Neurology	Parkinson’s disease	Dopamine enhancer	Suppression of glucagon synthesis and stimulation of insulin release in response to oral glucose load [320]	Hypoglycemia (?)
Istradefylline	Neurology	Parkinson’s disease	Adenosine (A2A) receptor antagonists	Potential for relevant impairment of intestinal glucose absorption (amelioration of non-fasting glycemia) [321]	-
Pimavanserin	Neurology	Parkinson’s disease	Serotonin (5-HT2A) receptor inverse agonism	Mitigation of appetite, delaying gastric emptying, weight loss [322]	-
Safinamide	Neurology	Parkinson’s disease	MAO-B reversible inhibitor	-	Potential affection of insulin secretion, apoptosis of β-cells (hyperglycemia and risk of new-onset T2D) [323]
Deutetrabenazine, Tetrabenazine	Neurology	Huntington’s disease	VMAT2 reversible inhibitors	Neutral effect on glucose and metabolic parameters [324]	Slight weight gain [325]
Methylphenidate, Lisdexamfetamine, Atomoxetine	Psychiatry	ADHD (adults)	Noradrenaline and dopamine reuptake inhibitors	Antidepressant effect, significant improvement of eating disorders, weight loss, improved glucose control [326]	Gastrointestinal discomfort, weight loss, or inability to gain weight
Buprenorphine	Neurology	Addictions	Opiate (mu) receptor partial agonism	Restriction of sugar consumption, caloric intake, and weight loss [327]	Constipation, nausea, and vomiting
Lofexidine	Psychiatry	Addictions	Central adrenergic (α2) receptor agonism	-	Enhancement of glucagon secretion, reduction in insulin secretion, lipolysis, gluconeogenesis, hyperglycemia [328]
Naltrexone	Psychiatry	Addictions	Opiate (mu) receptor antagonism	Relevant attenuation of impulsive eating and purging behaviors, weight loss, improvement of glucose control in patients with diabetes [329]	Constipation, nausea
Buprenorphine/Naloxone	Psychiatry	Addictions	Combining partial agonism and antagonism on opiate (mu) receptor	Restriction of sugar consumption, caloric intake, and weight loss [327]	Constipation, nausea, and vomiting

* Beneficial and detrimental effects are intended as mutual effects of concomitant medications on disease-related outcomes (e.g., in patients with T2D and PD, beneficial or harmful neurologic effects have been described for antihyperglycemic drugs, and vice versa). Abbreviations: AMPK = AMP-activated protein kinase; RNA = ribonucleic acid; PPAR = peroxisome-proliferator-activated receptor; DPP = dipeptidyl peptidase; GLP-1 = glucagon-like peptide-1; SGLT2 = sodium-glucose (co)transporter 2; USP30 = ubiquitin-specific protease 30; COMT = catechol-O-methyltransferase; MAO = monoamine oxidase; VAMT = vesicular monoamine transporter; ADHD = attention-deficit/hyperactivity disorder.

## 6. Conclusions and Future Perspectives

Dopamine signaling is involved in the fine regulation of several physiologic mechanisms that control cerebral functions, cognition, eating behaviors and reward, maintenance of glucose balance, and retinal, renal, and cardiovascular homeostasis. Impaired dopamine synthesis, metabolism, or activity is associated with neurological and psychiatric diseases, impaired glucose metabolism and T2D, and diabetes-related chronic complications. Evidence suggests that reinforcing dopamine signaling has a therapeutic role in T2D. The therapeutic implication should be better investigated in patients with dopamine-related disorders, such as PD, HD, addictions, and ADHD, as they are exposed to an increased risk of T2D, indicating the existence of a cross-link among these conditions. Understanding the potential interaction between pharmacological interventions in such disorders may ameliorate the management of patients, but specific trials are needed to confirm the therapeutic potential of certain medications. Last, emerging evidence suggests that dopamine imbalance is involved in developing chronic diabetes-related complications, and targeting dopamine metabolism would have the rationale for diagnostic and therapeutic purposes, as found in diabetic retinopathy.

## Figures and Tables

**Table 1:** Summary of mechanisms of dopamine-mediated modulation of β-cell activity, insulin, and glucagon secretion [6,7,8,9,10,11,12,13,14,15,16,17,18,19,20,21,22,23,24,25,26,27,28,29,30,31,32,33].

Mechanism	Effect	Consequences
Interference with insulin-containing grain trafficking (Dopamine-containing vesicles)	Blunt insulin release	Improvement in insulin sensitivity (e.g., insulin-resistant, obese patients) Deterioration of glucose control (non-insulin-resistant patients)
Impaired intra-pancreatic dopamine catabolism (Monoaminoxidases)	Catecholamine-induced (alpha and D_2_/D_3_ receptors) suppression of insulin synthesis and secretion	Hyperglycemia
Meal-induced intestinal synthesis of dopamine	Anti-incretin effect	Blunt insulin response after meal and post-prandial hyperglycemia
Enhancement of alpha-cell activity (High-dose dopamine)	Glucagon secretion	Fasting hyperglycemia (hepatic gluconeogenesis and glycogenolysis)
Suppression of prolactin release	Suppression of prolactin-induced insulin release	Hyperglycemia
Reduction in growth hormone	Amelioration of insulin release and peripheral insulin resistance	Improvement in glucose control (e.g., acromegaly)

Table 1 summarizes the mechanisms by which dopamine affects β-cell activity, insulin, and glucagon secretion. Each mechanism is associated with specific effects and potentially relevant clinical consequences in terms of the progression of diabetes and deterioration of glucose control.

## Abbreviations

L-DihydrOxyPhenylAlanine = L-DOPA; Dopamine Receptor = DR; Ventral Tegmental Area = VTA; Nucleus Accumbens = NAc; Prefrontal Cortex = PC; Amygdala = AMY; Parkinson’s Disease = PD; Type 2 Diabetes (mellitus) = T2D; Heart Failure = HF; Glucagon-Like Peptide-1 Receptor Agonists = GLP-1RAs; Sodium-Glucose (co)Transporter 2 Inhibitors (SGLT2is); Central Nervous System = CNS; Insulin Receptor Substrate 2 = IRS-2; Advanced Glycation End Products = AGEs; Advanced Glycation End Product Receptor = RAGE.
